# Editorial: Synthetic Biology of Yeasts for the Production of Non-Native Chemicals

**DOI:** 10.3389/fbioe.2021.730047

**Published:** 2021-08-11

**Authors:** Farshad Darvishi, Mark Blenner, Rodrigo Ledesma-Amaro

**Affiliations:** ^1^ Department of Microbiology, Faculty of Biological Sciences, Alzahra University, Tehran, Iran; ^2^ Research Center for Applied Microbiology and Microbial Biotechnology (CAMB), Alzahra University, Tehran, Iran; ^3^ Department of Chemical and Biomolecular Engineering, University of Delaware, Newark, DE, United States; ^4^ Department of Bioengineering, Imperial College London, London, United Kingdom; ^5^ Imperial College Centre for Synthetic Biology, Imperial College London, London, United Kingdom

**Keywords:** synthetic biology, yeast, metabolic engineering, bulk and fine chemicals, biotechnology, *Yarrowia lipolytica*, *Saccharomyces cerevisiae*

Yeasts are now considered attractive microbial cell factories for the production of a wide range of bulk and fine chemicals, including biofuels, pharmaceuticals, agrochemicals, fragrances, additives, pigments, and so on. Yeasts are easy to manipulate and scale-up and have a short generation and production time. However, metabolic engineering of yeasts is needed to make robust cell factories that produce the desired chemicals at high titers, rates, and yields. Synthetic biology techniques such as computational tools for metabolic modeling and pathway design, synthesis and assembly of standardized DNA parts, powerful genome editing methods and optimization of synthetic pathways, have been developed to improve the metabolic engineering of yeasts and to construct novel pathways for the production of non-native chemicals in a faster and more reliable manner without any additional metabolic burden.

This topic focuses on approaches of rewiring metabolism in yeasts to produce non-native chemicals, overcoming potential bottlenecks and on the identification of key challenges and future research trends. A short description of the articles included in this topic can be found below.

Meng et al. provided an overview of the latest research progress in the use of CRISPR/Cas9 systems in genome editing with a focus on the applications in synthetic biology of *Saccharomyces cerevisiae*.

Kim et al. reviewed synthetic pathways diverging from the distinctive cellular metabolism of bioethanol-producing *S. cerevisiae* and biodiesel-producing *Yarrowia lipolytica* to guide future directions for product-specific engineering strategies for the sustainable production of non-native chemicals.

Yocum et al. summarized the colocalization strategies of enzymes, including enzyme scaffolding, construction of synthetic organelles, and organelle targeting, in metabolic and synthetic pathways of yeast to ensure sufficient carbon flux towards the desired product.

Bradley et al. reviewed the recent progress in the biosynthesis of plant-derived natural products in yeasts, especially medically relevant halogenated alkaloids. The halogenated alkaloids are rare in nature. The introduction of halogenated substrates or halogenation enzymes enables the production of non-natural halogenated chemicals in yeast.

Arnesen et al. engineered *Y. lipolytica* for the production of terpenoids, including mono, sesqui-, tri-, di- and tetraterpenoids. They provided the first report for the production of β-farnesene and the highest reported titer of valencene (sesquiterpenoid) in *Y. lipolytica*. Furthermore, platform strains produced limonene, squalene, 2,3-oxidosqualene, or β-carotene.

The platform strains can be used for the evaluation of terpenoid biosynthetic pathways and it is a good starting point for constructing efficient cell factories of terpenoids.

López et al. produced β-ionone and β-carotene in engineered *S. cerevisiae*. The multiple expression of heterologous genes producing β-ionone and β-carotene were examined. Then, they carried out the tuning of the expression conditions to improve the production of β-ionone and β-carotene in the engineered *S. cerevisiae*, which resulted in the highest production of β-ionone in this yeast. Finally, a genome-scale metabolic model of *S. cerevisiae* was used to better understand β-ionone production in the engineered yeast and propose strategies to further enhance β-ionone titers.

Lajus et al. reported the production of poly-D-lactic acid (PDLA) in an engineered strain of *Y. lipolytica*. First, they identified and interrupted the pathway for lactic acid consumption in this yeast. Then, the heterologous pathway for PDLA production was introduced into the engineered strain. After that, PDLA homopolymer accumulated in the cells with the highest reported amount of produced PDLA *in vivo* so far.

Foo et al. generated strains of *S. cerevisiae* with the highest reported cytosolic aliphatic aldehyde and alkane/alkene production from fatty acyl-CoA by protein engineering of a fatty acyl-CoA reductase to alter its activity and metabolic engineering of *S. cerevisiae*.

Hambalko et al. reported the production of a fatty alcohol mixture by expressing bumble bee reductases in *Y. lipolytica*. A mixture of aliphatic unbranched fatty alcohols with eight or more carbons is naturally found in bumble bee as sex pheromones. A high titer and yield of fatty alcohols were obtained in the engineered strains by functional expression of bumble bee reductases from *B. lapidarius* (*Blap*Far) and *B. lucorum* (*Bluc*Far).

Park et al. engineered *Y. lipolytica* to produce ω-hydroxy palmitic acid from glucose using evolutionary metabolic engineering and synthetic FadR promoters for cytochrome P450 expression. They demonstrated *de novo* production of ω-hydroxy palmitic acid in batch fermentation containing nitrogen-limited media.

Overall, the collected works in this Research Topic present fabulous examples of advanced yeast metabolic engineering using synthetic biology in order to produce non-native chemicals of industrial interests.

Despite these exciting advances, the successful translation of non-native production of chemicals in yeast into the industrial practice is still limited. Just a few non-native chemicals are produced by yeasts on a commercial scale using metabolic engineering and synthetic biology tools. One of the major bottlenecks is making robust yeast strains to produce the chemicals at high titer, rate, and yield. The synthetic biology tools to construct synthetic pathways and rewire metabolism in yeasts are actively being developed to overcome these barriers.

Further advances in strain engineering technologies and computational guided synthetic biology are expected to streamline the strain creation process, but much work still need to be done. The creation of chassis strain, enriched in a particular type of products or their precursors can accelerate the number of successful stories. Interestingly, the type of yeasts being explored for bioproduction has expanded massively in recent years and we now see more and more examples of chemicals being made in non-*S. cerevisiae* microbes, as we can see in this issue. These non-conventional and non-model yeast hold great potential for overcoming the challenges associated with commercializing biotechnology when combined with the nascent and accelerating pace of synthetic biology tools across yeast platofrms.

In summary, there is a great potential in yeast biotechnology to address current sustainable manufacturing challenges and the scientific community is working hard and moving the field in the right direction.

